# Differential Binding of IgG and IgA to Mucus of the Female Reproductive Tract

**DOI:** 10.1371/journal.pone.0076176

**Published:** 2013-10-02

**Authors:** Kelly M. Fahrbach, Olga Malykhina, Daniel J. Stieh, Thomas J. Hope

**Affiliations:** Department of Cell and Molecular Biology, Northwestern Feinberg School of Medicine, Northwestern University, Chicago, Illinois, United States of America; Columbia University, United States of America

## Abstract

Cells of the endocervix are responsible for the secretion of mucins, which provide an additional layer of protection to the female reproductive tract (FRT). This barrier is likely fortified with IgA as has previously been shown in the gastrointestinal tract and lungs of mice. Mucus associated IgA can facilitate clearance of bacteria. While a similar function for IgG has been proposed, an association with mucus has not yet been demonstrated. Here we find that IgA and IgG are differentially associated with the different types of mucus of the FRT. We observed that while both IgA and IgG are stably associated with cervical mucus, only IgG is associated with cervicovaginal mucus. These findings reveal that antibodies can bind tightly to mucus, where they can play a significant role in the fortification of the mucus barriers of the FRT. It may be possible to harness this interaction in the development of vaccines designed to protect the FRT mucosal barriers from sexually transmitted diseases such as HIV.

## Introduction

Mucus is part of the natural barrier system of the female genital tract. It is a viscous hydrocolloid of associated and entangled mucins and other secreted proteins and acts as a lubricant, physical barrier, and trap for microbes [1]. Cervical mucus (CM) is generated by the combination of secreted mucins produced by goblet cells within the crypts of the cervix and cell associated mucins shed from the epithelial surface. Mucins 5AC and 5B are examples of secreted/gel-forming mucins responsible for the main structure of mucus in the female reproductive tract (FRT) [2], [3].

Previous work to determine the structure of CM has utilized electron microscopy to give detailed views of the mucus network and physical constraints for diffusion of macromolecules throughout mucus [4]–[6]. This revealed that the mucins form a meshwork that allows the unhindered diffusion of small compounds, while providing a barrier for larger pathogens such as bacteria. The detection of mucin genes in CM has been defined in more detail through in situ hybridization [7]. However, all the components of mucus have not been defined, therefore, we cannot yet discern with which factors pathogens interact. As the mucus migrates towards the cervical canal the secreted mucins combine with proteolytically liberated cell surface mucins released from the columnar epithelial cells lining the cervix. CM continues to move through the cervical canal toward the vagina and as it mixes with vaginal fluids and secretions it becomes cervicovaginal mucus (CVM) [8]. Due to mixing of these fluids, CVM is typically less viscous than CM and therefore may have different structure and protein composition.

The mucus barrier in the FRT may be fortified by the presence of antibodies, which can bind pathogens and facilitate their trapping into the network of mucins, as was initially suggested in the 1980’s [9]–[13]. For example, it is known that Muc2, highly expressed in the digestive tract, binds IgA through interactions mediated by secretory component (SC) of the secreted IgA dimer [14]. Interactions between SC and IgA in pulmonary mucus, where Muc2 is not present, have been shown to play a critical role in the clearance of bacteria [15], [16]. A mucin related protein that associates with the Fc portion of IgG, FcGBP, can also interact with Muc2, however, Muc2 is not typically associated with the FRT [17], [18]. Similar interactions may take place between IgA and mucins in saliva, including Muc5B, which is also in cervical mucus [19]. Interactions between mucins and IgG would allow IgG, the most abundant immunoglobulin (Ig) in the FRT, to facilitate pathogen retention in mucus [20]. The trapped pathogens would then be cleared as the mucus is shed. Little is known about the interactions of IgA and IgG with cervical mucus, however, published studies to detect the potential interaction of exogenous IgG and IgA failed to detect any stable interactions [21].

To gain additional insights into the interaction of IgA and IgG with mucus we examined the potential interaction of endogenous IgG and IgA with CM and CVM using several different experimental approaches. Examining cryosections of CM and CVM samples by immunofluorescent staining we were able to detect overlapping, yet distinct patterns of IgA and IgG in samples of CM, CVM, and in endocervical tissue suggesting potential binding of Igs to mucus. IgG and IgA interactions were shown to be stable within the CM network utilizing both short term photobleaching studies and long term dialysis experiments. In contrast, only IgG was observed to have a stable interaction with CVM. Understanding these differences and how Igs bind to mucus can contribute to our understanding of Ig function and potentially facilitate vaccine development.

## Results

### Visualization of CM and CVM structure by immunofluorescence

We obtained CM from consented donors during one of their regular gynecological visits and CVM was collected by consented donors through self-sampling with a soft cup without a visit to a physician (IRB# Stu25456). Samples were collected from non-menopausal women. Donor samples were obtained without knowledge of menstrual cycle, however, samples collected while the donor was menstruating were not used in imaging experiments. Previous work to identify localization of mucins in the FRT utilized mRNA or immunohistochemistry [22]–[24]. Paraffin embedding of tissue, however, requires multiple levels of fixation and harsher staining techniques that could modify structure of the mucus network [25]. Fixation followed by paraffin embedding often leads to dehydration of the sample, which could greatly affect the hydrocolloid nature of mucus. To better preserve the samples in their native structure we froze aliquots of CM and CVM in Optimal Cutting Temperature media (OCT) and then cryosectioned for immunofluorescence staining. A donor-matched control slide of tissue or mucus stained with secondary antibodies alone and imaged with the same exposure settings as slides receiving primary and secondary antibodies was included in all imaging sessions. Intensity of signals on slides receiving both primary and secondary antibodies were normalized to the control slides to detect true signal over background.

**Figure 1:**
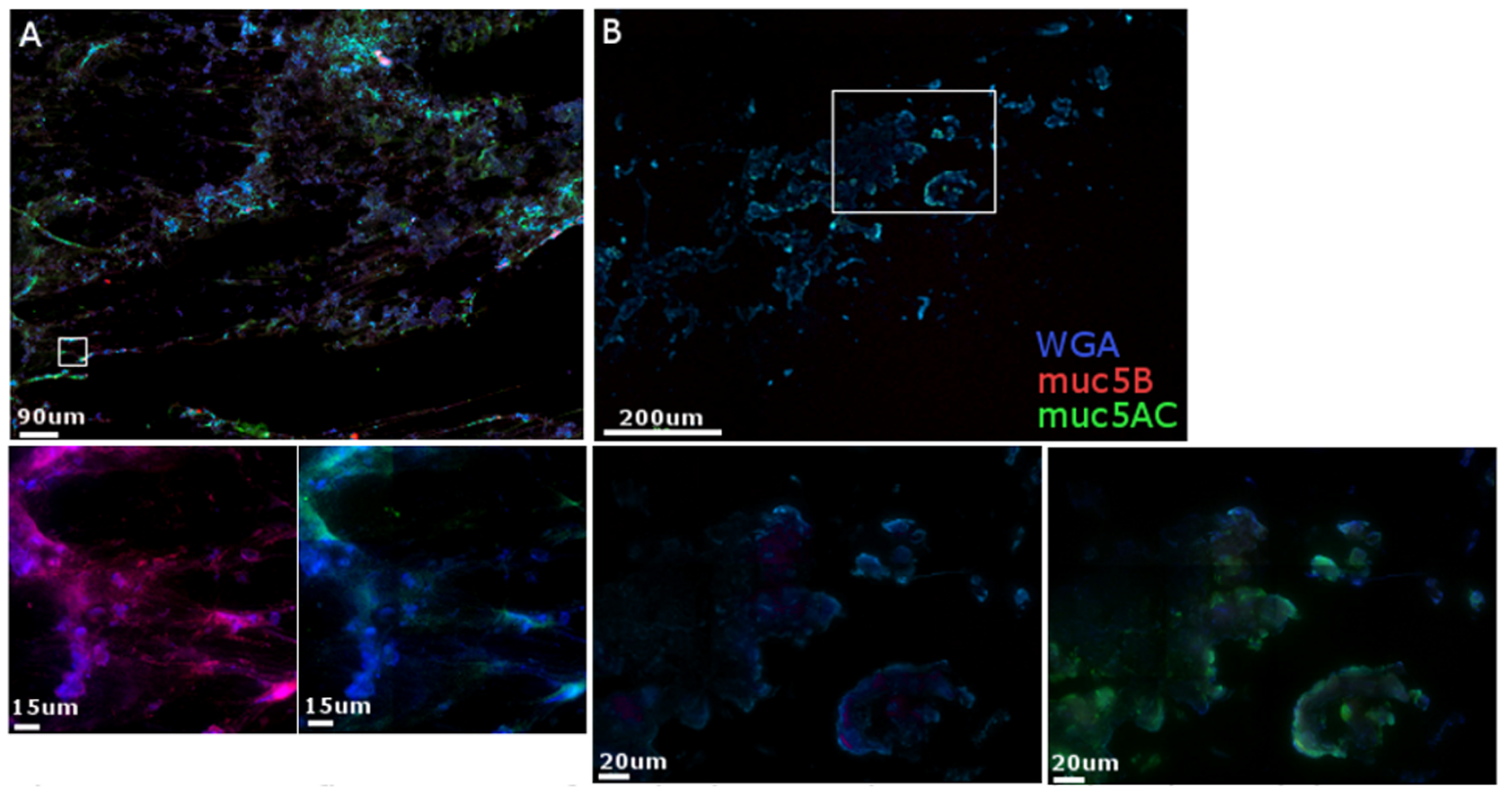
Immunofluorescence of mucins in CM and CVM. CM (A) and CVM (B) were frozen, cryosetioned, and fixed for immunofluorescent staining. All sections were stained for WGA (blue), mucin5AC (green), and mucin5B (red). Upper panels are images acquired with a 20X (A) or 10X (B) lens. Lower panels were acquired with a 100X lens.

We stained with wheat germ agglutinin (WGA), which nonspecifically binds polysaccharides and would give us a view of the overall structure of the mucus network. We also stained for the secreted mucins 5AC and 5B using antibodies specific for each. Low magnification images allowed us to observe that CM varied in structure from elongated fibers, small bodies, and larger globular clusters (Figure 1A, top). Over the course of samples from five different CM donors we consistently found an even distribution of mucin 5B along mucus fibers and among the small and large globules. Mucin 5AC was somewhat similar among the larger clusters of CM, but was not as apparent along the stretched out fibers. This can be seen in more detail in the smaller panels acquired at a higher magnification (Figure 1A, bottom). The overall structure of CVM differed from CM and appeared to have more globular portions than elongated fibers (Figure 1B). The levels of mucin 5AC were low, however, that which was detected was largely on the outer edges of mucus structures. Unlike CM, mucin 5B was not detected in CVM samples from three different donors. These experiments demonstrate that we can identify components of mucus through immunofluorescence and that there are differences in structure and protein content between CM and CVM.

### Detection of Igs in CM and CVM

**Figure 2:**
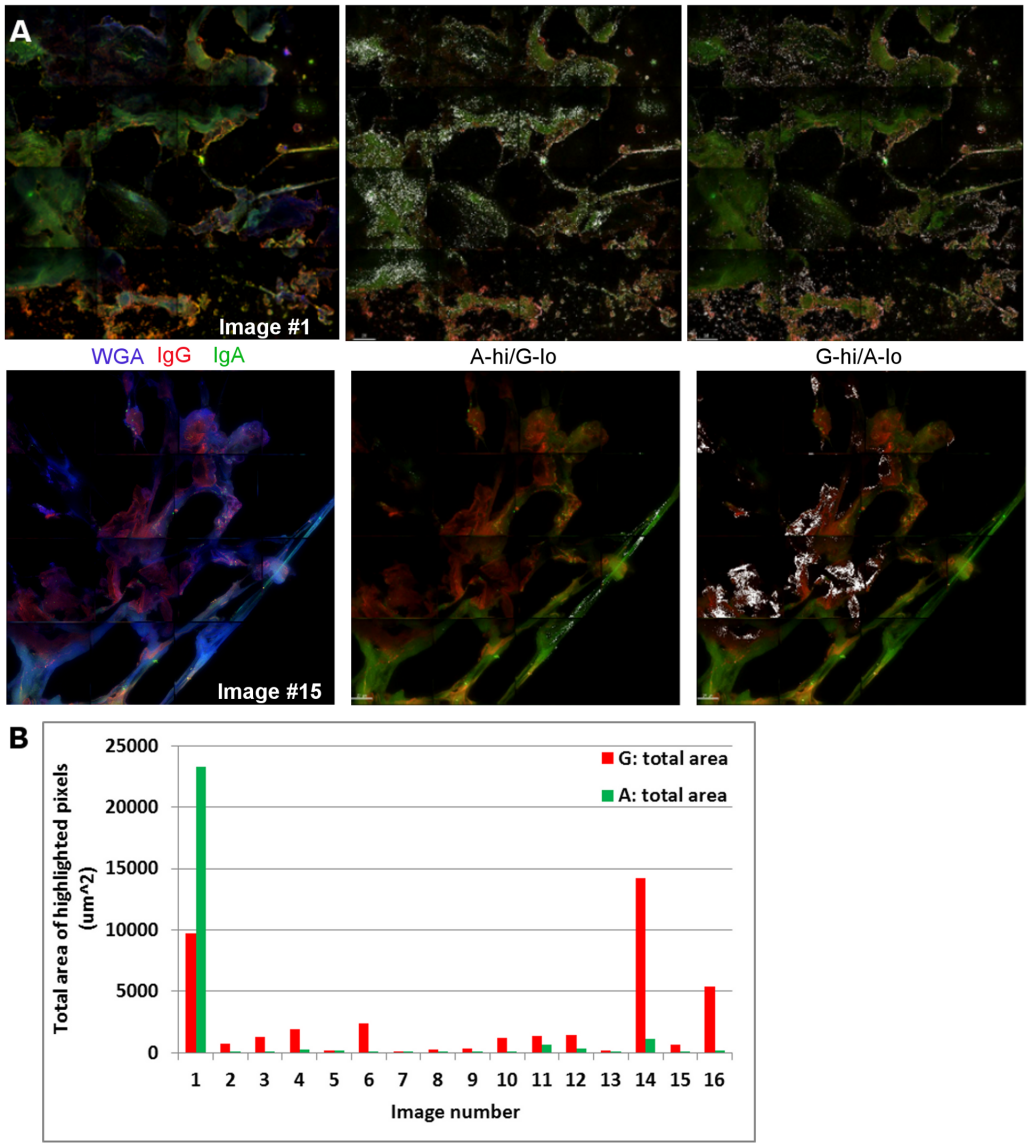
Ig expression in CM. (A) CM was stained for IgA (green) and IgG (red). Left column shows Ig expression with WGA (blue). White pixels highlight regions of A-hi/G-lo (middle column) and G-hi/A-lo (right column) staining. (B) Graph of total area of highlighted pixels for IgG and IgA dense regions.

**Figure 3:**
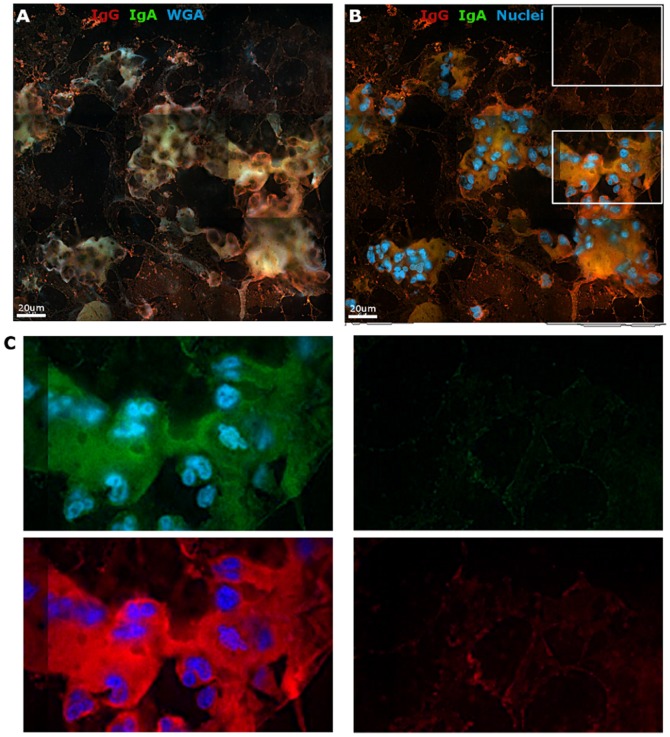
Immunofluorescence of IgG and IgA in CVM. Frozen CVM was stained for IgG (red) and IgA (green) and imaged with a 100X lens. (A) CVM also stained with WGA (blue). (B) Same field of view in (A) with nuclei now shown in blue. (C) Zoom in of white boxes in (B) to show separated IgA (top) and IgG (bottom) stains.

After detection of mucin proteins by indirect immunofluorescent staining of mucus cryosections we looked for the presence of IgG and IgA. Secondary controls were used in these samples as above. Samples receiving both primary and secondary antibodies were normalized to these controls to remove background signal from the tissue itself. We did not observe any background due to the staining antibodies themselves interacting with components of the mucus. In CM samples we noticed that IgG and IgA both had unique areas of accumulation and also some areas of overlap (Figure 2A). To gain information as to how much the two Igs overlapped, we performed colocalization analysis on the images. The Pearson coefficient scores the level of colocalization. A large proportion of the two Igs overlap throughout the image, which is indicated by a Pearson value approaching 1.0 (Table 1). However, in our images we observed portions of IgG and IgA that deviated from this and had more distinct expression. We defined areas where the IgG expression was high and IgA expression was low (G-hi/A-lo) and vice versa (A-hi/G-lo) based on pixel intensity (representative example in Figure S1). These highlighted areas are depicted as white pixels on the images (Figure 2A, middle and right images). Not only is there variability in mucus structure between samples, but also within an individual sample. To get a sense of this variability a total of 16 panel images were acquired across five different CM samples. Both the number of unique clusters of white pixels and total area of all of these regions are shown in Table 1 (samples lettered A-E). The representative images shown in Figure 2A are samples A1 and E2 from this table. Apparent in the images is that when A-hi/G-lo pixels are highlighted they are diffuse or in the interior areas of the CM network. In contrast, the G-hi/A-lo regions are seen more often on the edges of the mucus. The total area of all highlighted pixels for IgA and IgG in each image is shown as a graph in Figure 2B, where it is evident that the total area of G-hi/A-lo pixels is higher in all but one of the images. Ig expression patterns were also examined in CVM. Samples were stained for IgG, IgA, and WGA as with CM and we found that both Igs associated with the CVM network (Figure 3A). However, a confounding factor of CVM is the abundant presence of shed vaginal epithelial cells. These cells are much more prevalent in CVM and not CM due to CVM being associated with the vaginal epithelium where squamous epithelial cells are constantly shed. A stain for cell nuclei readily reveals the high number of epithelial cells present in CVM (Figure 3B). Magnification of an area with cells and a cell-free area demonstrates that regions of CVM where epithelial cells were not present revealed staining primarily for IgG (Figure 3C). In contrast, IgA staining was weak in the cell-free areas of CVM. The variances observed in both the enumeration with CM and images from CM and CVM suggest that IgG and IgA are integrated into the mucus network differently and could interact with components of the network via different means. The trends observed for IgG and IgA expression and their unique staining patterns suggest differences in Ig incorporation into and associations with mucus.

**Table 1:** Quantification of IgG and IgA dense regions in CM images.

Image number	Ghi/Alo total number ^1^	Ghi/Alo total area ^2^	Ahi/Glo total number ^1^	Ahi/Glo total area ^2^	Pearson coefficient
A1	5568	9678	9859	23293	0.5857
A2	304	686	0	0	0.8745
A3	102	1283	0	0	0.9059
B1	666	1925	89	234	0.7814
B2	109	182	83	138	0.8395
C1	477	2377	0	0	0.8593
C2	0	0	0	0	0.8485
C3	198	286	0	0	0.855
C4	213	318	0	0	0.8000
D1	439	1157	10	48	0.7193
D2	484	1326	183	605	0.8726
D3	756	1403	133	308	0.7006
D4	60	129	0	0	0.9144
E1	3159	14244	28	1114	0.7408
E2	259	651	5	14	0.7402
E3	733	5390	48	206	0.8182

Regions of high IgG and low IgA (Ghi/Alo) and Ahi/Glo detection were highlighted as described in the results and materials and methods. Data from five samples from different CM donors is shown, donors labeled A-F. Panel images acquired for each donor are numbered with each donor letter.

1: total number indicates the total number of unique highlighted areas counted in the image.

2: total area indicates the total area of all highlighted pixels in the image.

### Detection of Igs in endocervical explants

**Figure 4:**
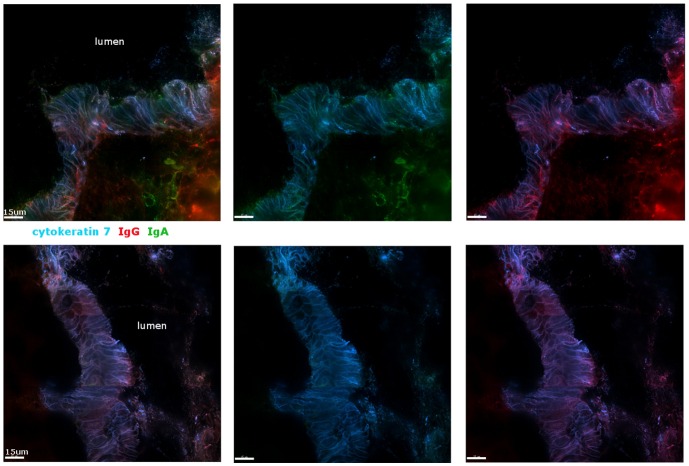
Detection of immunogloblulins in endocervical explants. Sectioned explants of endocervix were stained with antibodies to detect IgA (green) and IgG (red). Cytokeratin 7 (blue) was also stained to identify the layer of columnar cells. Each row represents a different donor sample. Images were acquired with a 100X lens as a panel and then stitched together. Second and third columns have red or green color removed, respectively, from image in first column.

We next performed immunofluorescent staining for IgG and IgA in endocervical tissue in archival control explants which were cultured for 4 hours. If Igs were associating with mucins we may be able to detect Ig protein along the columnar cells of endocervix where cell associated and secreted mucins are abundant. Cryosections of explants from seven different donors were stained for IgG and IgA, normalized to controls stained with secondary antibodies alone, and examined via immunofluorescence. Cytokeratin 7 was used as a counterstain to confirm the examined regions were columnar epithelial cells and reveal tissue structure of the endocervix. Figure 4 displays two representative samples of Ig stained endocervix. Both of these samples show detection of IgG along the surface of the columnar cells and also accumulating in mucus in the lumen. In contrast, we observed more variance with detection of IgA. Four out of seven samples had IgA visible along the epithelium of the endocervix (Figure 4, representative sample in top row). The remaining three samples had low or very minimal detectable IgA above background controls (Figure 4, representative sample in bottom row). The variability in detection of IgA could be due to differences in native flora of the patients or differences in menstrual cycle as to when the samples were collected [26]. The detection of IgG and IgA at the surface of the endocervical epithelium supports evidence for cells within the endocervix as a source of Ig secretion; and it allows for Igs to interact with mucus being secreted from the same location.

### FRAP analysis of Igs in mucus

**Figure 5:**
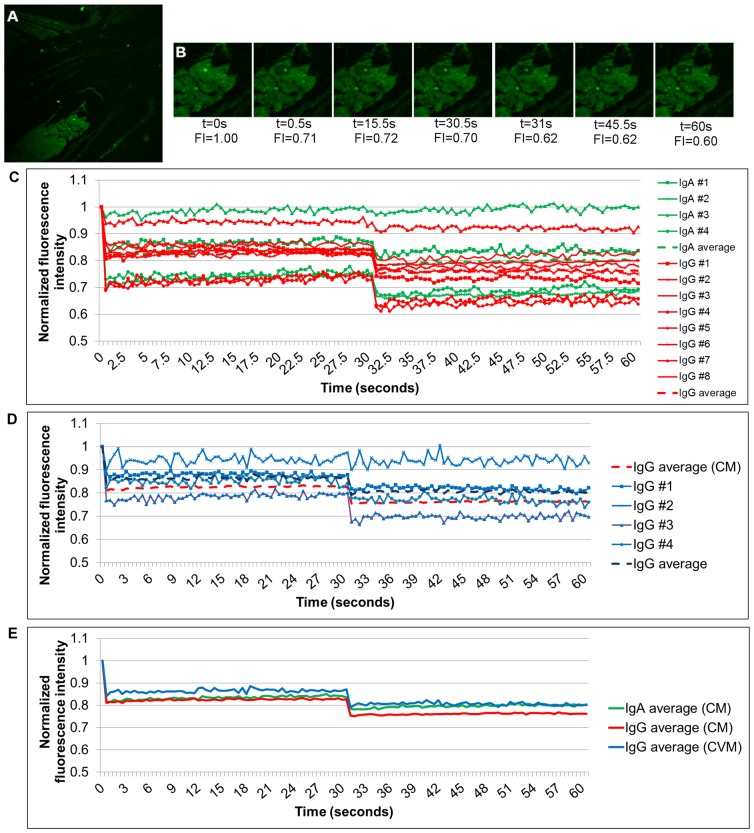
FRAP in mucus. (A) 100X image of fluorescently labeled IgG in CM. (B) Zoomed in ROI from (A) at select timepoints. Images were acquired every 0.50ms over the course of 1 minutes, with a rebleach after 30 seconds. Fluorescence intensities (FI) after normalization are shown. (C) Graph of normalized intensities of photobleached ROIs in IgG or IgA labeled CM. (D) Graph of normalized intensities of photobleached ROIs in IgG labeled CVM. CM average from (C) is included for comparison. (E) Average values from other graphs redisplayed.

While we were able to visualize IgG and IgA within the mucus network, it remained unclear whether these Igs were stably or transiently associated with mucus fibers. To answer this question we performed fluorescence recovery after photobleaching (FRAP) imaging on mucus samples where either endogenous IgG or IgA had been fluorescently labeled green with Fab’s or conjugated antibodies, respectively, and added to fresh mucus samples. The addition of labeled primary antibodies or antibody fragments allowed us to identify endogenous IgA and IgG as opposed to adding exogenous fluorescently labeled IgG and IgA that may not be able to incorporate into the already formed mucus structure/network. The structures observed with labeling of endogenous IgG in native samples was reminiscent of that observed in the fixed cryosectioned samples described above (Figure 5A). Small regions of interest (ROI) were identified and subjected to photobleaching. Our primary interest was to determine if there was any short-term recovery of signal in the bleached region that would indicate diffusion of labeled Ig or binding of free label to this ROI. Figure 5B illustrates a representative example of one of the timelapses where IgG was labeled in CM. It is clear where the photobleaching takes place, visible as a rectangle of decreased fluorescence representing the region of interest. The graph depicts normalized signal throughout the time course of multiple samples (Figure 5C).

Averages of normalized data are shown in Figure 5E. Both the images and graphical data reveal a lack of recovery of both IgG and IgA in CM and IgG in CVM over the 30 second time period of analysis for the majority of samples. The average FRAP profiles for IgG in CM and CVM and IgA in CM are all very comparable in their degree of photobleaching and lack of recovery. The labeled Ig associated with the mucus does not diffuse out of the ROI to allow other labeled, unbleached Ig to move into its position. We also do not observe any additional binding by free label present in the sample, suggesting there are no available binding sites for the labeled molecules. The presence of freely diffusing anti-IgG/IgA label was confirmed with attempts to bleach background regions of the mucus sample where no defined structure was observed (data not shown). We were unable to bleach these regions, indicating a constant presence of freely diffusing label. Therefore, the lack of recovery of signal in bleached ROIs of IgG and IgA labeled mucus suggests that both of these endogenous Igs can have short term stable interactions with the mucus network over the course of at least 1–2 minutes.

We performed these same experiments on CVM samples to determine if there was any degree of IgG or IgA binding to CVM. Due to CVM being composed of more vaginal fluids than CM, however, we found that the fluorescent labeling of endogenous IgG in native CVM was less apparent and more challenging to image. The samples we were able to image and analyze demonstrated a smaller extent of photobleaching compared to CM, suggesting larger amounts of unbound IgG (Figure 5D and 5E). However, we did not see 100% recovery of signal in CVM, revealing there is also an immobile fraction of IgG present in its network. In contrast to CM, it was not possible to detect any IgA in the CVM above background signal.

To gain further insight into the degree of immobility of IgG, a subset of these samples were subjected to repeated bleaches of the same ROI. This would result in all labeled molecules in the ROI becoming more fully photobleached and, therefore, make it easier to detect if there were any lower levels of mobile IgG that were replenishing the region. We observed a drop in signal of the ROI after each subsequent bleach as the immobile fraction became more photobleached (Figure S2). After repeated photobleaching of the same region it was possible to detect a small increase in IgG signal return to the region over time in two of the three samples analyzed. When these intensity values were compared to those after the first bleach, however, the slight recovery was not statistically significant. Since we did not observe recovery of photobleached regions in IgG or IgA in labeled CM and of IgG in CVM, but were unable to detect bound IgA in CVM, these photobleaching experiments suggest there may be differential association of Igs with CM and CVM.

### Dialysis of Igs in mucus

**Figure 6:**
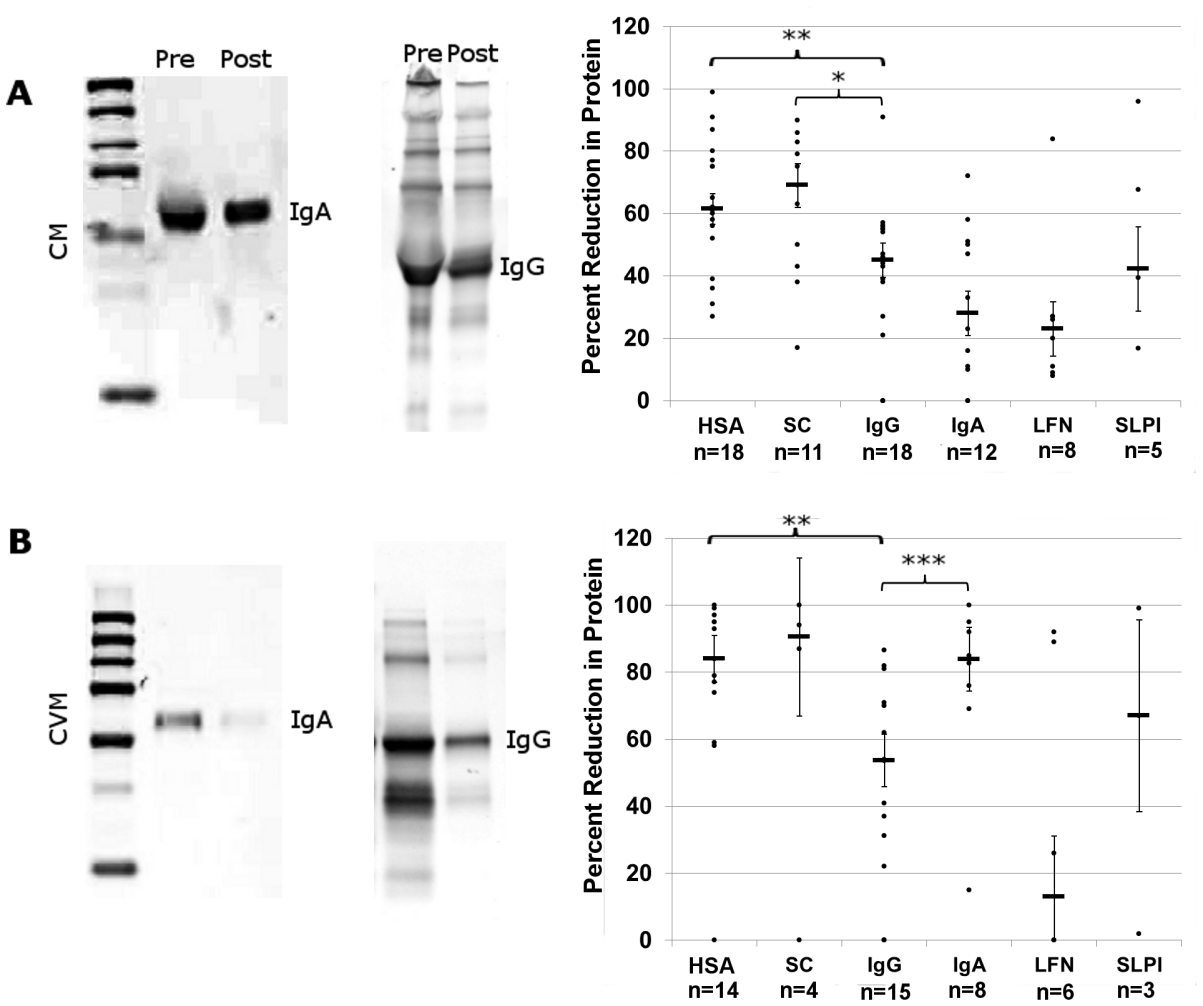
Dialysis of CM and CVM. CM (A) and CVM (B) were dialyzed through a 700 kDa filter (Post) and loaded onto gels for western blotting with pre-dialyzed mucus (Pre). Blots were stained for IgA or IgG. Various proteins known to be components of mucus were identified pre-and post- dialysis, the intensities of bands quantified as described, and the percent reduction graphed. P-value <0.01 indicated by a *, <0.005 by a **, and <0.001 by a ***. Abbreviations: HSA: human serum albumin, SC: secretory component, LFN: lactoferrin, SLPI: secretory leukocyte protease inhibitor.

The photobleaching studies described above revealed short term interactions of Igs with mucus. To examine the potential stable, long-term association of Igs we performed 48 hour dialysis on fresh CM or CVM samples from multiple donors against PBS using a membrane with a cutoff of 700 kDa. Pores of this size readily allow the Igs to exit the sample chamber, while the much larger macromolecular complexes contained within the mucin network would be retained. Samples of CM and CVM collected both pre- and post-dialysis were subjected to western blotting. We detected a robust presence of both IgA and IgG after dialysis of CM, indicating the existence of a bound fraction for both of the Igs (Figure 6A). Dialysis of CVM revealed different results, as we observed a statistically significant loss of IgA signal post-dialysis while IgG was retained in the sample chamber (Figure 6B). This correlates with the FRAP data described above, in the inability to detect a fixed fraction of IgA in CVM. There is some reduction in IgG post-dialysis in both CM and CVM, but a dense band is still present suggesting a portion of IgG is bound to proteins within both.

Many mucus samples were also examined by western blot for human serum albumin (HSA), secretory component (SC), IgG, IgA, lactoferrin (LFN), and secretory leukocyte protease inhibitor (SLPI), which are commonly found in mucus and served as internal controls [proteomic analysis of CM, unpublished data, T.J.Hope] (Figure S3). The intensity of each protein band was quantified and the data graphed as percent reduction of each protein (Figure 6). Other than LFN and IgG, it appears that all the proteins examined dialyze more readily from CVM than CM. The compiled data reveal that the median for IgG and IgA reduction in dialyzed CM was at approximately 50% and 30%, respectively. This further supports the presence of two populations of IgG and IgA, immobile and diffusing, as first suggested by the short term FRAP studies. Unlike with CM, dialysis of CVM revealed a striking difference in IgA levels (Figure 6B). The median reduction of IgG in CVM, as in CM, was around 50%, while the median reduction of IgA in CVM approached 90%. While there was no significant difference between the dialysis of IgG in CM versus CVM, the difference in percent reduction of IgA between CM and CVM is significant (p-value of 0.000828). This data reveals the presence of a fraction of IgG and IgA within CM and of IgG in CVM that is tightly bound within the mucus network and that there is a loss of protein association in CVM versus CM.

## Discussion

Our results use both imaging and biochemical techniques to identify a previously undefined fraction of IgG that is bound to mucus of the FRT (CM and CVM) and of IgA that is bound to CM, but not CVM. We report data supporting these stable interactions through the use of three different experimental approaches: fixed immunofluorescence, FRAP, and dialysis. First, immunofluorescent imaging of cryosections of CM and CVM allowed us to observe differences in IgG and IgA incorporation in CM and CVM in a novel manner (Figures 2 and 3).

Next, FRAP of endogenous IgG and IgA in mucus allowed us to assess kinetic interactions of Igs with mucus over a short period of time. We discovered that fluorescently labeled Igs were unable to diffuse into bleached regions of mucus, suggesting that a fraction of both IgG and IgA are tightly bound to the CM network (Figure 5). We also had indications that IgG and IgA differ in their incorporation into CVM, as an immobile phase of IgG was found, but IgA could not even be detected.

Finally, we developed a third set of analyses to examine the long term association of Igs with mucus over days (versus minutes as in the FRAP experiments). We discovered that a fraction of IgG was stably associated to both CM and CVM to a similar degree as protein was still detected even after two days of dialysis of either type of mucus. However, IgA was lost after the 2 day dialysis of CVM (Figure 6). These findings indicate that both IgG and IgA in CM and IgG in CVM can remain bound to the mucus for extended periods.

Results from these three assays demonstrate that Igs and other proteins have different degrees of association with CM and CVM. This suggests the movement of mucus from the CM-containing upper tract to CVM-containing lower tract can act as a mechanism for delivery of proteins to different areas of the FRT. For example, a decrease in mucin5B is observed in CVM compared to CM (Figure 1). Immunofluorescent staining of CM demonstrated the presence of both IgG and IgA, each with its own unique pattern within the network (Figure 2). IgA tended to be spread evenly over the network and IgG densely accumulated on the edges of the CM network. This suggests that there is not an arbitrary association of Igs with mucus and that there are more specific protein:protein interactions occurring. In CVM IgA was identified largely around the cells themselves, which is not surprising as secretory component can transcytose through these cells to deliver IgA to the lumen of the cervix (Figure 3). The presence of epithelial cells in CVM samples confounded their detailed analysis, however, there was decreased detection of IgA over IgG in cell-free areas of CVM. The decreased IgA detection in CVM and unique patterning of Ig association may be a pivotal aspect of the mechanism of how components of mucus are delivered from the upper to lower FRT.

IgG and IgA were both detected and found to have stable fractions in CM in the FRAP experiments, however, we could not detect IgA in these samples of CVM (Figure 5). This further supports the hypothesis that the stability of IgA association with mucus changes between CM and CVM. The stable interactions of IgG in mucus observed here contrasts previously published data which did not detect any stable association [21]. However, these previous studies utilized exogenous fluorescently labeled IgG added to mucus while this current work studied IgG endogenous to CM and CVM. If the IgG binding sites in the mucus were already saturated with endogenous Igs, there would be no ability for the exogenous IgG to bind to the mucus. This suggests that the binding of IgG to mucus is a consequence of a limited number of high affinity binding sites for IgG present in the mucus.

Dialysis of CVM revealed that IgA readily diffused out of the mucus. This is in contrast to the very stable IgG interaction with CVM which was detected in both the dialysis and FRAP assays. In addition to the drastic loss of IgA over two days of dialysis, other mucus related proteins (HSA, LFN, SLPI) were also lost more readily in CVM compared to CM (Figure 6). It is important to consider that the turnover of mucus in the FRT is on the order of 24 hours. Therefore, IgG retention for 2 days could potentially trap pathogens long enough for clearance. In contrast, the loss of IgA protein via dialysis in CVM suggests that it almost completely exists as a diffuse/loosely bound protein in CVM. The association in CM but not CVM of IgA and other factors further supports that mucus secreted within the cervix may function as a delivery system for factors to the vaginal vault, where they are released into the lumen. Furthermore, the stable binding of IgG can be targeted as a means of generating specific immune responses in the FRT.

The binding of IgA to mucus has been previously reported for pulmonary and gastrointestinal mucus [14], [16]. Binding of IgG to mucus of the gut has been shown to occur through mucin proteins directly and also through adapter molecules such as FcGBP [27] [17]. FcGBP has many similarities to the mucin proteins, has been detected in various forms of mucus, various tissues (including the FRT), and has been suggested as a factor important for mucus structure [17], [27], [28]. The presence of proteins in mucus that can directly interact with Igs would clearly be advantageous in the trapping and clearance of pathogens. In fact, mucin 2 within the upper layers of colonic mucus is believed to be a primary mechanism in preventing invading pathogens to penetrating further into lower layers closer to cells that can be infected by the pathogens themselves [17]. Based on differences we observed between CM and CVM we speculate that the loss of IgA in CVM versus CM may be due to changes in its interactions with mucins or other proteins associated with mucus. For example, either IgA may be released from mucins of CVM or the mucins themselves are being degraded to allow diffusion of IgA from the network.

Studies on the respiratory tract demonstrate that IgA and IgG concentrations vary in different types of mucus collected throughout the tract [29]. This largely affected the efficacy of vaccines based on how they were administered and whether there was an IgG or IgA based immune response. Our findings here describing the tight binding of IgG to mucus of the FRT, and lack of IgA binding in CVM, have important implications to understanding immune function protecting these mucosal surfaces. Therefore, learning more about the distribution of these Igs in CM and CVM can play a critical role in the development of vaccines against mucosal pathogens.

Utilizing IgG bound to mucus of the FRT could be a viable alternative in the development of vaccines to prevent acquisition of pathogens such as HIV in women. The presence of pathogen binding IgG in mucus could enhance pathogen trapping by tethering it to the mucus network. The trapped pathogen would then be shed before it could reach the cellular barriers of the FRT. Current vaccines readily generate virus binding, but non-neutralizing IgG. Antibodies tethered to mucus, however, could readily trap pathogens shed during heterosexual transmission. Trapping via antigen binding interactions with mucus-bound Ig could effectively neutralize the pathogens. Such binding would essentially make them uninfectious if it could not physically diffuse to mucosal epithelial barriers and underlying target cells.

The data presented here lays the groundwork for subsequent experiments that will more specifically examine Ig interactions with mucus and proteins within the mucus network. Now that we are aware these interactions and differences in mucus exist, a quantitative analysis of both CM and CVM during different stages of the menstrual cycle is warranted to determine if there are significant changes in mucus composition based on cycle. It can also be determined if the differences in IgA binding between CM and CVM varies throughout the cycle. To potentially harness this interaction to function as a vaccine it will be important to determine the nature of the tight binding mucus:Ig interaction. Specifically, it is critical to understand how the Igs, mucins, and other potential accessory molecules are interacting with one another and become incorporated into the mucus network. Understanding these mechanisms may allow vaccine responses to be directed to accumulate in the mucus of the FRT where they could function to provide a level of protection in the fight against infection.

## Methods

### Ethics Statement

All patients gave written informed consent. The protocol for collection of samples was approved by Northwestern University Feinberg School of Medicine Institutional Review Board (Study #25456).

### Collection of mucus samples

Samples of cervical mucus were collected from the cervical os by physicians during a regular gynecological exam. Mucus was obtained by aspirating with a mucus collection device, Mucat™ (Sepal Reproductive Devices, Bostom, MA, USA). Cervicovaginal mucus samples were collected via self-sampling by the patients themselves with a commercially available Instead Softcup® (EvoFem, Inc., San Diego, CA, USA). The cup was inserted for a minimum of 3 hours by self-reported mid-cycle donors. All mucus samples were delivered to the laboratory immediately after collection and prepared for FRAP or dialysis within 48 hours of receipt or cryopreserved.

### Indirect Immunofluoresence staining

Mucus samples were frozen in optimal cutting temperature (OCT) media and stored at –80°C. Cervical tissue was isolated from consented patients undergoing hysterectomies at Northwestern Memorial Hospital and explants of endocervix preserved in OCT media (Study #00025456). Endocervix and mucus samples were cryosectioned, fixed, and stained with combinations of the following antibodies as described in the results: mucin 5AC (BD Pharmingen), mucin 5B (Invitrogen), IgA (Invitrogen), IgG (BD Pharmingen), cytokeratin 7 (Dako), WGA, or Hoechst. Secondary antibodies used were Oregon Green (Molecular Probes, Life Technologies) or Rhodamine RedX anti-mouse (Jackson Immunoresearch). Alexafluor Zenon kits 594 and 647 (Molecular Probes, Life Technologies) were also used to directly label primary antibodies.

### Microscopy of fixed samples

Indirect immunofluorescent images were acquired on Deltavision deconvolution microscopes. The different magnification lenses were used as noted in the results and figure legends. Panel images were acquired and stitched together using SoftWorx software. A control slide of tissue or mucus stained with secondary antibodies alone was included in all imaging sessions and panels were acquired on these slides with the same exposure settings as the fully stained slides. Intensity of signals on slides receiving both primary and secondary antibodies were normalized to the control slides to detect true signal over background.

### Analysis of IgG and IgA in mucus

During data acquisition the exposure settings were adjusted so that the maximum intensities of IgG and IgA were similar. Fluorescence intensities were normalized to control slides and the degree of colocalization of the fluorophores used to stain for IgG and IgA was calculated using a colocalization tool in SoftWorx software. G-hi/A-lo areas were defined by selecting pixels that had intensities greater than twice the mean fluorescence of the IgG stain and less than the mean fluorescence of the IgA stain. Comparable analysis was performed for marking pixels on each image that were considered A-hi/G-lo. Images with white pixels were then analyzed with ImageJ software to calculate both the total number of uniquely highlighted regions and the total surface area of highlighted pixels for each image.

### FRAP

CM or CVM was incubated with either component A (Fab’s) of Zenon AlexaFluor human IgG 488 labeling reagent or with mouse anti-human IgA labeled with Zenon AlexaFlour mouse IgG 488 kit (Life Sciences, Molecular Probes) to label endogenous IgG or IgA. After 1–2 hours of labeling, samples were immediately imaged on a Zeiss laser scanning confocal microscope in Northwestern University Cell Imaging Facility. Frames were acquired every 500ms over the course of a minute, with the sample rebleached after 30 seconds. The intensity of the bleached region was calculated using ImageJ FRAP profiler (Macbiophotonics version), and photobleaching of the overall sample was taken into account during intensity calculations. The reported intensity values were calculated by normalizing the recorded fluorescent intensity of the ROI at each timepoint to the initial scan (which was valued at 1). For repeated bleaching experiments the ROI was bleached a total of four times with images acquired every 500ms after each bleach. The act of photobleaching ranged from 5–25 seconds each time is occurred.

### Dialysis and western blots

Samples of CM or CVM were spun in a centrifuge to remove cellular debris from the sample. Within four days of receipt, 50 ul of mucus was placed into a dialyzer (The Nest Group, Southborough, MA) and samples were dialyzed for two days in PBS at 4°C. Polycarbonate membranes of 700 kDa or cellulose acetate membranes of 5 kDa molecular weight cut offs (The Nest Group, Southborough, MA) were used.

Pre-dialyzed and post-dialyzed samples were reduced and denatured in Laemmli’s buffer by boiling for 3 minutes and run on a SDS-PAGE gel. Goat anti-IgG (Sigma-Aldrich, U.S.), rabbit anti-IgA (Abcam, Cambridge, MA), rabbit anti-serum albumin (Rockland, Gilbertsville, PA), mouse anti-secretory component (GeneTex, Irvine, CA), goat anti-lactoferrin (Novus Biologicals, Littleton, CO), and mouse anti-SLPI (Santa Cruz Biotechnology, Santa Cruz, CA) were used as primary antibodies. Donkey anti-goat IRDye 800CW, donkey anti-rabbit IRDye 680LT, goat anti-mouse IRDye 800CW, donkey anti-mouse IRDye 680LT (LI-COR Biosciences, U.S.) were used as secondary antibodies. Odyssey Infrared Imaging System (LI-COR Biosciences, U.S.) was used to detected immunostained protein bands. Protein bands were quantified using Image Studio Ver1.1. The percent reduction of each protein was calculated by taking the percentage of protein detected in the post-dialyzed condition compared to the pre-dialyzed condition, and then subtracting from 100. One-tailed, unequal variance T test was performed on the quantified protein bands to determine significance.

## Supporting Information

Figure S1 **Representative colocalization graph of bottom row of images in** **Figure 3** **.** Axes denote intensity of signal for each pixel of the fluors used to stain IgA and IgG. Pixels selected to mark G-hi/A-lo areas on the image are marked off by the box. Pixels with 2X the mean of IgG and less than the mean of IgA were selected for highlighting regions of dense IgG and low IgA levels.
(JPG)

Figure S2 **Repeated photobleaching of IgG in CM.** Endogenous IgG was fluorescently labeled in CM as described in the Results and Materials and Methods. A region of interest (ROI) was photobleached every 30 seconds over the course of 2 minutes (timecourse lasted 3-4 minutes due to additional time to perform bleaches). The intensity of IgG fluorescence in the ROI at the initial scan pre-bleach was set at 1 and subsequent values normalized to this.
(JPG)

Figure S3 **Dialysis of control proteins in CM and CVM.** Mucus was subjected to dialysis through a 700 kDa filter (Post700K), or a 5 kDa filter (where noted Post5K). Aliquots of CM (A) and CVM (B) pre and post-dialysis were run on a western blot and proteins probed with the noted antibodies. Abbreviations: HSA: human serum albumin, SC: secretory component, LFN: lactoferrin, SLPI: secretory leukocyte peptidase inhibitor.
(TIF)
