# Supplementary figures and images for: Differential Binding of IgG and IgA to Mucus of the Female Reproductive Tract
Source: PLoS One. 2013 Oct 2;8(10):e76176. doi: 10.1371/journal.pone.0076176 (PMC3788792; doi:10.1371/journal.pone.0076176)

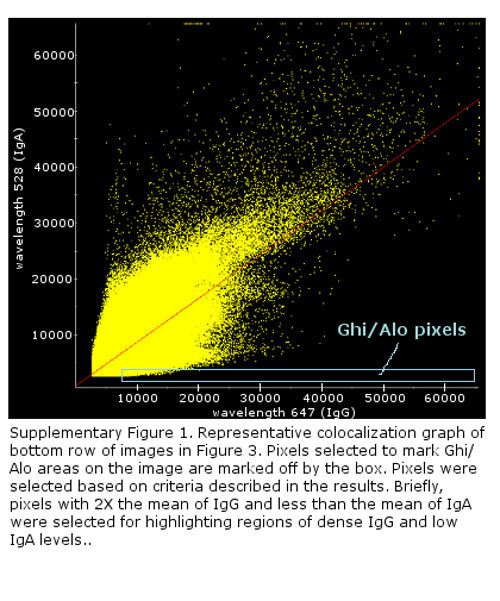

Supplement: Figure S1 — Representative colocalization graph of bottom row of images in Figure 3 . Axes denote intensity of signal for each pixel of the fluors used to stain IgA and IgG. Pixels selected to mark G-hi/A-lo areas on the image are marked off by the box. Pixels with 2X the mean of IgG and less than the mean of IgA were selected for highlighting regions of dense IgG and low IgA levels. (JPG) [file pone.0076176.s001.jpg]

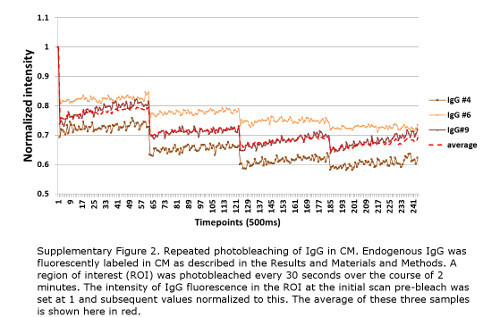

Supplement: Figure S2 — Repeated photobleaching of IgG in CM. Endogenous IgG was fluorescently labeled in CM as described in the Results and Materials and Methods. A region of interest (ROI) was photobleached every 30 seconds over the course of 2 minutes (timecourse lasted 3-4 minutes due to additional time to perform bleaches). The intensity of IgG fluorescence in the ROI at the initial scan pre-bleach was set at 1 and subsequent values normalized to this. (JPG) [file pone.0076176.s002.jpg]

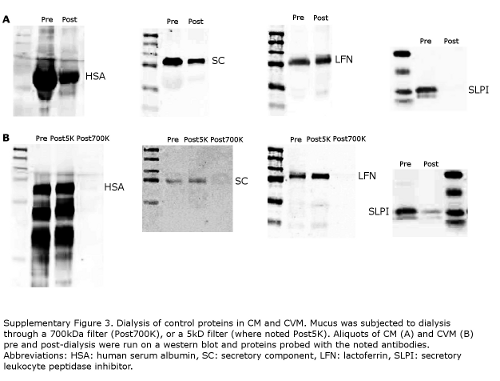

Supplement: Figure S3 — Dialysis of control proteins in CM and CVM. Mucus was subjected to dialysis through a 700 kDa filter (Post700K), or a 5 kDa filter (where noted Post5K). Aliquots of CM (A) and CVM (B) pre and post-dialysis were run on a western blot and proteins probed with the noted antibodies. Abbreviations: HSA: human serum albumin, SC: secretory component, LFN: lactoferrin, SLPI: secretory leukocyte peptidase inhibitor. (TIF) [file pone.0076176.s003.tif]
